# High-Speed Videoendoscopy and Stiffness Mapping for AI-Assisted Glottic Lesion Differentiation

**DOI:** 10.3390/cancers17081376

**Published:** 2025-04-21

**Authors:** Magdalena M. Pietrzak, Justyna Kałuża-Olszewska, Ewa Niebudek-Bogusz, Artur Klepaczko, Wioletta Pietruszewska

**Affiliations:** 1 Department of Otolaryngology, Head and Neck Oncology, Medical University of Lodz, 90-419 Lodz, Poland; magdalena.pietrzak@umed.lodz.pl (M.M.P.); ewa.niebudek-bogusz@umed.lodz.pl (E.N.-B.); 2 Institute of Electronics, Lodz University of Technology, 90-924 Lodz, Poland; justyna.sujecka@dokt.p.lodz.pl (J.K.-O.); artur.klepaczko@p.lodz.pl (A.K.)

**Keywords:** glottic cancer, vocal fold stiffness, high-speed videoendoscopy, laryngotopography, Stiffness Asymmetry Index, machine learning, receiver operating characteristic, benign lesions, malignant lesions, kymography, amplitude asymmetry, phase difference of vocal fold oscillations

## Abstract

**Objectives**: This study evaluates the potential of high-speed videoendoscopy (HSV) in differentiating between benign and malignant glottic lesions, offering a non-invasive diagnostic tool for clinicians. Moreover, a new parameter derived from high-speed videoendoscopy (HSV) had been proposed and implemented in the analysis for an objective assessment of the vocal fold stiffness. **Methods**: High-speed videoendoscopy (HSV) was conducted on 102 participants, including 21 normophonic individuals, 39 patients with benign vocal fold lesions, and 42 with glottic cancer. Laryngotopographic parameter describing the stiffness of vocal fold (SAI) and kymographic parameters describing amplitude, symmetry, and glottal dynamics were quantified. Statistical differences between groups were assessed using receiver operating characteristic (ROC) analysis and lesion classification was performed using a machine learning model. **Results**: Univariate receiver operating characteristic (ROC) analysis revealed that SAI (AUC = 0.91, 95% CI: 0.839–0.962) and weighted amplitude asymmetry (AUC = 0.92, 95% CI: 0.85–0.974) were highly effective in distinguishing between normophonic and organic lesions (*p* < 0.01). Further multivariate analysis using machine learning models demonstrated improved accuracy, with the SVM classifier achieving an AUC of 0.93 for detecting organic lesions and 0.83 for distinguishing benign from malignant lesions. **Conclusions**: The study demonstrates the potential value of parameter describing the pliability of infiltrated vocal fold (SAI) as a non-invasive tool to support histopathological evaluation in laryngeal lesions, with machine learning models enhancing diagnostic performance.

## Simple Summary

Early detection of glottic cancer remains a challenge due to changes in vocal fold biomechanics. This study assessed the Stiffness Asymmetry Index (SAI), derived from high-speed videoendoscopy (HSV), as a quantitative marker of vocal fold stiffness for differentiating benign and malignant glottic lesions. Analysis of 102 participants revealed that SAI (AUC = 0.91, 95% CI: 0.839–0.962) and weighted amplitude asymmetry (AUC = 0.92, 95% CI: 0.85–0.974) effectively distinguished between normal and pathological vocal folds. Machine learning models further improved classification, with an SVM classifier achieving an AUC of 0.93 for organic lesion detection and 0.83 for malignancy differentiation. These findings highlight SAI as a promising non-invasive parameter for early glottic cancer detection, with machine learning enhancing diagnostic accuracy.

## 1. Introduction

Dysphonia is usually the first symptom of different disorders of the voice organ including organic lesions as well as functional or inflammatory disorders. The differentiation between possible causes of dysphonia is necessary for adequate treatment modalities. While in benign lesions the main goal is to obtain good quality of voice, the treatment of the malignant lesions is being planned mainly to obtain clear margins. Laryngeal cancer (LC) is one of the most common malignancies in the head and neck region and about 95% is laryngeal squamous cell carcinoma (LSCC) [1]. In the glottis, it usually appears on the mucosal surface of the free margin in the anterior 1/3 of the vocal fold. Early diagnosis of the LSCC is a key point to a complete cure. According to the 5-year survival rate which ranges from 78% in early-stage patients to 34% in advanced cases with metastatic involvement [2]. The histopathological examination remains a gold standard for the diagnosis of organic lesions. However, an effort is made to obtain a highly objective method for the examination before invasive procedures, which could support the diagnosis. Clinical evaluation of the larynx is the first step in the process. An essential point is the white light endoscopy (WLE) which guides treatment planning. However, WLE could be insensitive for early, superficial lesions. Therefore, many studies recommend combining WLE with narrow band imaging (NBI) which asses the vascular patterns [3,4]. European Laryngological Society published a descriptive guideline for vascular changes in lesions of the vocal folds. They pointed out the important value of NBI assessment in the vocal fold lesions. However, authors also stated that NBI examination results must remain as part of other findings e.g., mucosal vibration, vocal fold stiffness [5]. Objective assessment of the vocal fold stiffness had different approaches in time. Firstly it was assessed invasively and, therefore could not be implemented clinically as it caused scaring and required general anesthesia [6]. Then the non-invasive methods were developed based e.g., on the relationship between fundamental frequency (F0) and subglottic pressure (Ps) which allows the clinical use of this parameter [7,8]. According to the endoscopy in larynx, the mucosal wave is commonly assessed and its absence is partly a result of vocal fold stiffness. Commonly laryngovideostroboscopy (LVS) is used for the functional assessment of vocal fold oscillations. This method gives the illusory image of the vocal fold vibrations using the synchronization of strobe light with F0. However, it fails in aperiodic voices or patients who had problems with prolonged phonation, so mostly cases with severe dysphonia. High-speed videoendoscopy (HSV) overcomes some of those problems. This method allows us to obtain the real oscillations of the vocal fold, recording at more than 2000 fps. There are many methods for the objective assessment of HSV among others the laryngotophography (LTG). LTG is a method for the assessment of spatial characteristics. Fourier transformation is used to detect changing in the time brightness of pixels across HSV images [9,10]. In 2022 we proposed implementation of the LTG maps to calculate Stiffness Asymmetry Index (SAI) to provide a quantitative comparison of vibrations between affected and non-affected vocal folds [11].

The goal of the study is to propose the non-invasive approach for differentiation of benign and malignant glottic lesions using a newly proposed parameter describing the vocal fold stiffness (SAI) based on the laryngotopography (LTG) derived from the high-speed videoendoscopy (HSV). Moreover, to implement the new parameter (SAI) in the objective assessment of vocal fold oscillations to support the standard kymographic parameters describing the vocal fold symmetry, amplitude and phase difference with use of machine learning methods.

## 2. Materials and Methods

### 2.1. Study Group

Laryngeal HSV recordings (*n* = 102) of subjects hospitalized between March 2020 and March 2024 at the Department of Otolaryngology, Head and Neck Oncology, Medical University of Lodz, Poland was taken. The inclusion criteria for the study required that the vocal folds in the recordings were clearly visible during high-speed videoendoscopy (HSV) imaging. Cases where the epiglottis obstructed the anterior commissure, or the arytenoid cartilages obstructed the posterior vocal folds were excluded. The study cohort consisted of 21 normophonic individuals (healthy controls), 39 patients diagnosed with benign vocal fold lesions (such as polyps and cysts), and 42 patients with early malignant lesions (all diagnosed with T1 squamous cell carcinoma of the vocal fold).

According to sex and age in the group of 42 patients with malignant lesions there were 34 males, 8 females, aged from 44 to 91 years, with mean age of 68 years. In the group of 39 patients with benign lesions there were 17 males and 22 females aged from 23 to 77 years, with mean age of 50 years. Normophonic group of 21 patients consists of 7 males and 14 females aged from 22 to 68 years, with a mean age of 43 years.

The final diagnosis of all lesions was confirmed by histopathological examination of tissue specimens obtained from hypertrophic lesions. Patients with bilateral vocal fold lesions or advanced cancer affecting other structures (laryngeal ventricle, vestibular fold, contralateral vocal fold) were excluded from the study.

### 2.2. Equipment and Instrumentation

HSV recordings were carried out using a rigid, oval-shaped endoscope (Xion ϕ10.0, Berlin, Germany) with light input in Storz standard matched to a 4.8 mm fiber optic light cable. The HSV images of the larynx were registered with an Advanced Larynx Imager System (ALIS) manufactured by Diagnova Technologies (Wroclaw, Poland), equipped with an innovative endoscope laser light source (ALIS Lum-MF1) and laryngeal high-speed camera (ALIS Cam HS-1) with a CMOS image sensor with a color filter array (the classic Bayer filter) and a global shutter. Images were recorded 3200 frames per second at 416 × 416 pixels.

### 2.3. Endoscopic Procedure

The high-speed videoendoscopy (HSV) procedure was conducted under controlled conditions, with patients seated in a comfortable position and instructed to maintain stable phonation of the vowel “i” for the duration of the recording at a comfortable level of pitch and volume. The vocal fold movements were captured over a period of a few seconds during sustained phonation. The recordings were carefully reviewed to ensure that no other anatomical structures interfered with the laryngeal views (e.g., epiglottis or arytenoid cartilages obstructing the vocal folds).

### 2.4. Laryngotopographic (LTG) Analysis

Stiffness Asymmetry Index (SAI) was calculated using the laryngotopography (LTG) method. The Fourier Transform was applied on so-called brightness functions defined for every individual pixel within the glottal area. This function is simply a time-series of image pixel brightness values that periodically change due to the vocal folds motion, either obscuring or exposing the glottis lumen. Since the frame rate of the HSV camera used in the study (3200 fps) is much higher than the expected fundamental frequencies range of vocal folds oscillations, the reconstructed brightness functions can be reliably used for signal harmonic analysis. Specifically, Fourier analysis is used to decompose the brightness fluctuations of each pixel over time into frequency components. This enables us to:

#### 2.4.1. Identify Fundamental Frequency (F0) and Harmonic Components

By analyzing pixel intensity variations, we can determine the dominant vibratory frequencies of the vocal folds, distinguishing between normal and pathological oscillations.

#### 2.4.2. Map Spatial Vibratory Patterns

Fourier Transformation is applied across the entire glottic region, creating laryngotopographic maps, which visually represent areas of high and low vibratory activity. These maps are crucial for detecting non-vibratory regions, amplitude asymmetry, and stiffness changes associated with malignancy.

#### 2.4.3. Quantify Oscillatory Behavior in a Standardized Manner

FT-based spectral analysis allows for objective comparison of different glottic regions, reducing observer bias and providing numeric parameters that can be used in machine learning classification.

**Figure 1:**
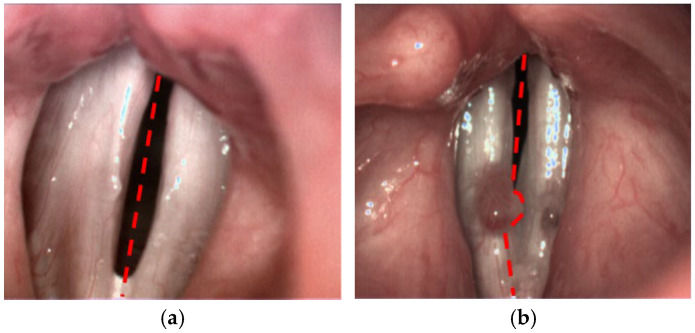
Different approaches for the partitioning of the glottis into left and right side: (**a**) using straight symmetry axis and (**b**) locally adapted boundary to avoid assigning right vocal lesion to the left side.

This method enabled the objective evaluation of the mucosal wave, amplitude asymmetry, and phase difference, with the goal of identifying significant differences between normal and pathological vocal fold movements. The SAI was calculated based on the LTG maps, allowing a quantitative comparison of the vibratory patterns of the affected vocal fold with the non-affected one. Detailed methodology is presented in another publication [12]. In comparison to the published version of the algorithm, for the purpose of the current study we have introduced one major improvement with respect to the lateral separation of the vocal folds. Previously, the user simply indicated the top and the bottom poles of the glottal gap. These two locations determined the symmetry axis of the glottis (see Figure 1a). In the pilot study, this approach ensured accurate division between left and right side. However, there are cases, where the lesion on one side obscures the vocal tissue on the other side, thus disturbing the estimation of the motion dynamics of the unaffected part. Therefore, we modified the procedure of SAI calculation through a more precise delineation of the boundary between the two sides, adapted to the local shape of glottal lesions (Figure 1b).

For the analysis we used short recordings of 625 ms therefore the movement of the endoscope tip is negligible. However, perspective distortion, although a well-recognized issue in endoscopic imaging, remains challenging to fully eliminate. Moreover, studies proved that there is an influence of angle tilt of the endoscope on the parameter values especially those describing the asymmetry [13]. A fundamental approach to mitigating this distortion in laryngoscopy involves ensuring optimal orientation of the endoscope tip, such that the optical axis of the camera is maintained perpendicular to the surface of the vocal folds. Additional strategies include the use of advanced image post-processing techniques to correct distortion effects, as well as the implementation of 3D imaging, which provides depth information and enables more accurate reconstruction of anatomical structures. These techniques are however still in the research and development phase.

Therefore, in this study, video sequences were meticulously acquired with careful attention to optimal endoscope positioning, ensuring reliable quantification of vocal fold dynamics. Furthermore, it is important to highlight that the SAI parameter exhibits inherent robustness to distortions arising from moderately deviated viewing angles. This parameter is calculated as the ratio of the relative vibrating areas of the left and right vocal folds calculated separately. When distortion is minimal, any apparent enlargement on one side effects both the vibrating and total fold areas proportionally, thereby preserving the accuracy of the SAI calculation.

### 2.5. Kymographic Analysis

The obtained recordings were analyzed with the aid of the Diagnova Technologies software DiagnoScope Specialist ver. 1.3 (Poland) dedicated for ALIS with additional modules. This software provides tools for both short- and long-term kymographic analysis and parameterization. While HSV is the primary imaging modality, our analysis is based on high-speed kymography, which is derived from HSV recordings. For the analysis the software automatically selected a part of the recording 625 ms length. Manual stabilization and centering of the image for each frame was not required, because, in such a short recording time (625 ms), the movement of the endoscope tip is negligible. After this the examiner marked five points on a single representative frame and based on this the vocal folds edges were identified semi-automatically along the whole length of the glottis. In case of any discrepancy the software allows to verify and adjust the automated detection by the examiner. We used the kymography which is a post-processing technique that extracts temporal vibratory information from HSV recordings, enabling quantitative assessment of vocal fold oscillations. In the final step, the software calculated a set of specific parameters to quantify vocal fold dynamics, including amplitude, asymmetry, and phase differences. Those parameters are presented in the Table 1. Their significance were mentioned in the ELS/UEP guidelines as important in the voice assessment [14]. A detailed description of the apparatus and software used is provided in another publication [15]. Detailed explanation with mathematical formulas please see the Supplementary S1.

### 2.6. Statistical Analysis

The objective of the statistical analysis was to assess the ability of each parameter to differentiate between benign and malignant patients, as well as distinguish both types of organic lesions from healthy individuals. Before performing group comparisons, we assessed the normality of all 39 kymographic parameters using the Shapiro-Wilk test (α = 0.05). Parameters that followed a normal distribution were analyzed using one-way ANOVA. Parameters that did not follow a normal distribution were analyzed using the Kruskal-Wallis test.

To determine which parameters significantly differed between the three patient groups (normophonic, benign, malignant), we applied: one-way ANOVA for normally distributed parameters; Kruskal-Wallis test for non-normally distributed parameters.

For parameters that showed a significant difference in the global ANOVA/Kruskal-Wallis test, we conducted Mann-Whitney U tests for pairwise comparisons: normophonic vs. benign lesions; normophonic vs. malignant lesions; benign vs. malignant lesions. Bonferroni correction was applied to adjust for multiple comparisons.

Six parameters were found to be significantly different across groups: Stiffness Asymmetry Index (SAI); Weighted Amplitude Asymmetry (AmplAsymWeighted); Average Amplitude Asymmetry (AmplAsymAvg); Amplitude Asymmetry (Middle Third) (AmplAsymAvg_2/3); Absolute Phase Difference (AbsPhaseDiffAvg); Weighted Absolute Phase Difference (AbsPhaseDiffWeighted)

For the most significant parameters, Receiver Operating Characteristic (ROC) analysis was conducted to evaluate their classification performance. To compensate for small sample size we had applied ROC analysis with boostrapping. In all simulations, number of bootstrap iterations was set to 1000. Dataset was resampled with replacement so that the size of the resampled data was equal to the original.

The mean metrics were averaged over bootstrap iterations. The 95% confidence bounds were found as 2.5 and 97.5 percentiles of the results obtained through the 1000 repetitions. In this analysis, the ROC curves are interpolated, as the TPR values in different bootstrap run were obtained for different FPRs. The ROC analysis was conducted in two stages: first, to differentiate normophonic patients from those with any organic lesions (benign and malignant), and second, to distinguish between benign and malignant lesions. The Youden Index, sensitivity and specificity with 95% confidence intervals were reported. We have added a definition of the Youden Index, which is used to determine the optimal cut-off threshold for binary classification in our ROC analysis. The Youden Index (J) is calculated as:

J = Sensitivity + Specificity − 1

Youden indexes are calculated for the mean ROC curves, but the threshold cutoff-values can only be given for the full original data. Otherwise, determination of a cutoff value is ambiguous. A higher Youden Index indicates a better balance between sensitivity and specificity, making it a useful metric for selecting the optimal diagnostic threshold for parameters such as SAI and amplitude asymmetry.

### 2.7. Machine Learning Model

To further examine the diagnostic capability of the parameters in the multivariate setting, a machine learning model was developed. The purpose of the study was to build a classifier model for discriminating patients according to the diagnosis (Norm, Benign, and Malignant). With the approach used, there is no need to make assumptions about the type of probability distribution of the data, and the discriminative power of parameters can be evaluated not only individually, but also in their subsets. For the classifier model we chose the Support Vector Machines (SVM) algorithm with the radial basis kernel function [16]. The model was then built using parameters identified as significant using an attributes selection method based on the so-called wrapper approach. The input data was the entire dataset—102 patients described by the 39 parameters listed with the descriptions in the Table 1.

The following analysis steps were performed:

1. Parameter standardization. For each parameter, the mean value and standard deviation were calculated. After subtracting the mean and dividing by the standard deviation, each parameter had mean = 0 and standard deviation = 1. Thus, parameters with a larger range of values had the same effect on the distance metric calculated between data vectors as parameters with a small range.
2. Selection of significant attributes. This step was performed using the sequential floating-forward search (SFFS) algorithm [17]. The exploration of the multidimensional parameter space in search of significant features (providing the best classification of the selected classifier) is done sequentially. First, a single parameter is found that minimizes classification error, and then more features are added or subtracted depending on their impact on the classification accuracy.
3. Training of the final classifier model. The SFFS algorithm terminates when further addition or removal of attributes does not improve classification quality. The final subset is used to develop and test a classifier model of multidimensional data vectors. The following SVM models have been developed:

- A model for the classification to all 3 classes simultaneously.
- A binary model to discriminate between Norm and any organic lesion
- A binary model to discriminate between Malignant and Benign lesions.

In each case, learning and testing of the models was done using 5-fold cross-validation. This means that the dataset was randomly divided into 5 parts. Learning was repeated 5 times, each time a different 4 parts were used to build the model, and testing was performed for the remaining fifth part. This is a major difference from the evaluation of threshold classifiers proposed in statistical analysis, in which the entire data set is used to determine the cutoff value. As common as this is in statistical analysis, the final metrics, such as sensitivity and specificity, are seemingly better than they might actually be if the same cutoff value were used for new patients. Therefore, for binary classifiers in addition to cross-validation, testing was also performed on the entire learning set to enable direct comparison with the results obtained previously.

Moreover, the training and testing of the classifiers described in step 3 were also conducted for the subset of features identified as significant in MCT tests. These experiments allow for the evaluation of the effectiveness of multivariate analysis based on machine learning in comparison to univariate statistical analysis

## 3. Results

### 3.1. Univariate Analysis

The kymographic parameters were compared across three distinct patient groups: normophonic individuals, benign lesion patients, and malignant lesion patients. The statistical comparison revealed six key parameters that differed significantly between all three groups, based on the U Mann-Whitney test (*p* < 0.01). Among these, the Stiffness Asymmetry Index (SAI) emerged as a critical factor, along with two parameters describing phase difference and three parameters quantifying amplitude asymmetry (Table 2). These findings suggest that these parameters provide strong differentiating power when evaluating different types of vocal fold lesions.

**Figure 2:**
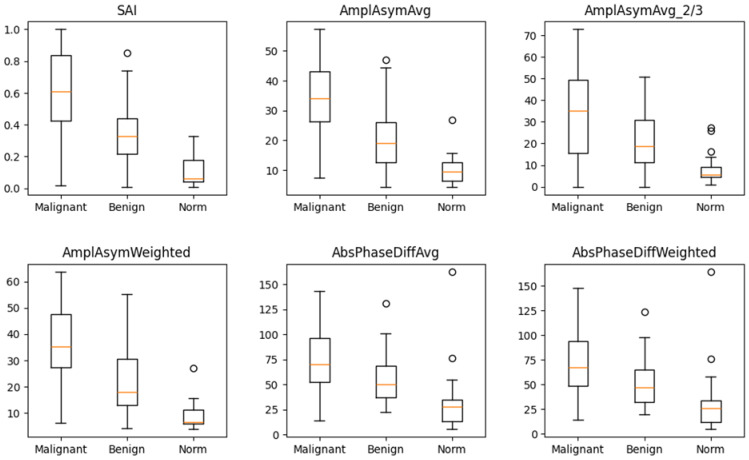
Box and whisker plots for six parameters which differ in a significant way simultaneously between normophonic patients, benign and malignant glottic lesion groups. Diagram presents median values of individual vibratory parameters in groups divided by diagnosis, specifically: norm, benign, and malignant. The dots correspond to an elevated value of parameters.

The box-and-whisker plot (Figure 2) visually illustrates the distribution of values for these six parameters in the three groups. The median values for these parameters differed significantly between normophonic subjects, benign, and malignant lesion groups. However, no single parameter was able to clearly distinguish between all three groups on its own.

To further assess the discriminatory power of these parameters, ROC curve analysis was conducted. This analysis was performed in two stages: first, to differentiate normophonic individuals from patients with any organic lesion (benign and malignant), and second, to distinguish between benign and malignant lesions.

**Figure 3:**
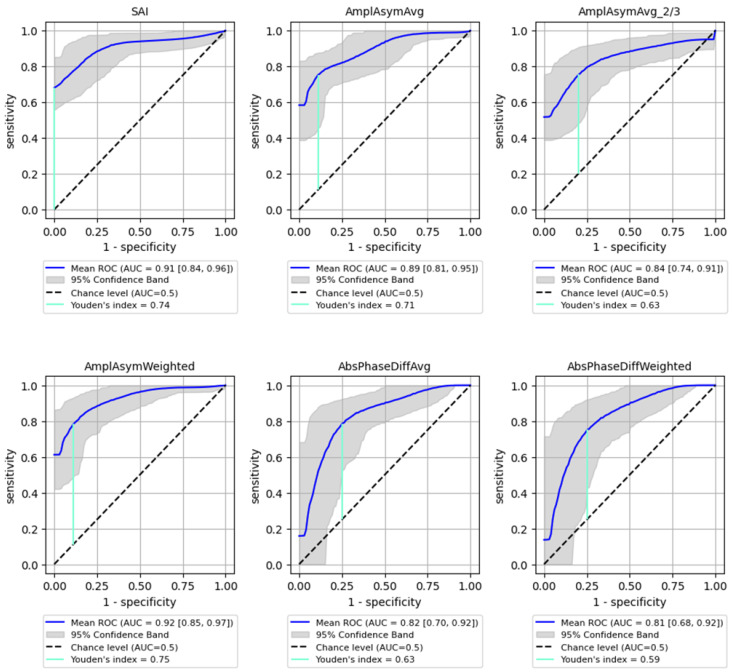
ROC curves with bootstrapping for the six best-performing vibratory parameters differentiating normophonic subjects from patients with any organic lesion of the glottis (both benign and malignant) (blue line). *X*- and *y*-axis present sensitivity and 1-specificity of points on the curve. The black line presents an AUC value of 0.5; the aquamarine line presents the value of the Youden index.

The best performance in differentiating normophonic subjects from patients with any organic lesion (benign and malignant) was observed for the weighted amplitude asymmetry (AmplAsymWeighted), which yielded an AUC of 0.92 (95% CI: 0.85–0.974) with 83% sensitivity (95% CI: 0.697–0.952) and 92% specificity (95% CI: 0.765–1.0, *p* < 0.0001) indicating excellent discriminative ability. SAI also showed strong performance, with an AUC of 0.91 (95% CI: 0.839–0.962), 83% sensitivity (95% CI: 0.623–0.966) and 90% specificity (95% CI: 0.737–1.0, *p* < 0.0001). Altogether six parameters mentioned above reached AUC values above 0.8, with varying sensitivity and specificity as shown in Figure 3.

The Youden Index was calculated for each parameter, providing a cut-off threshold for detection of organic lesions. The sensitivity and specificity values associated with these cut-off points are summarized in Table 3, indicating high sensitivity and specificity for most parameters, particularly for SAI (0.83 sensitivity, 0.9 specificity). Sensitivity (True Positive Rate): Measures the proportion of actual positive cases correctly identified (i.e., how well the model detects malignant lesions); Specificity (True Negative Rate): Measures the proportion of actual negative cases correctly classified (i.e., how well the model avoids false positives in detecting malignancy).

**Figure 4:**
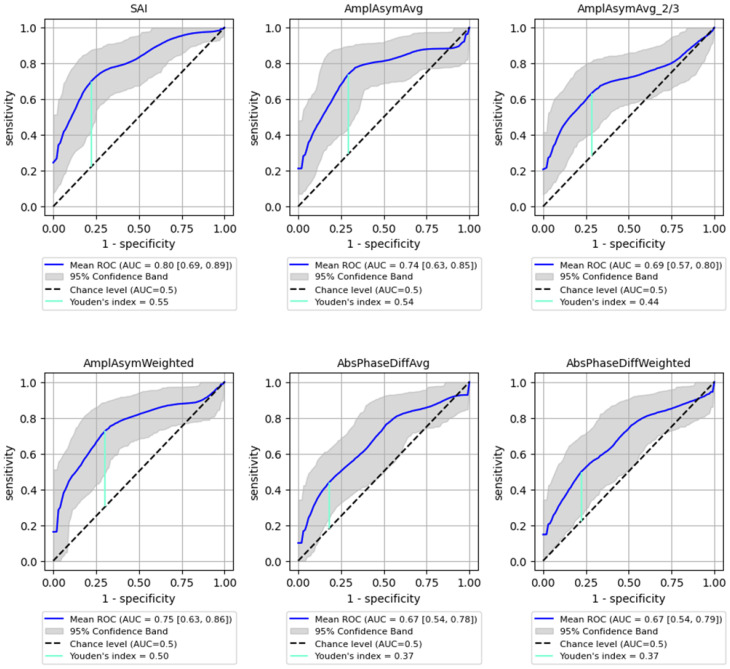
ROC curves with bootstrapping for the six best-performing vibratory parameters differentiating benign from malignant lesions of the glottis (blue line). *X*- and *y*-axis present sensitivity and 1-specificity of points on the curve. The black line presents an AUC value of 0.5; the aquamarine line presents the value of the Youden index ending on the curve.

ROC analysis for distinguishing between benign and malignant lesions showed a generally lower AUC than for detecting the organic lesions. However, SAI remained a significant parameter, achieving an AUC of 0.8 (95% CI: 0.692–0.886) with 73% sensitivity (95% CI: 0.540–0.913) and 81% specificity (95% CI: 0.621–0.955, *p* < 0.0001), while Amplitude Asymmetry parameters (e.g., AmplAsymAvg and AmplAsymWeighted) reached AUC values around 0.75. These values are substantial but lower than those obtained for detecting any organic lesion. Detailed results are shown in Figure 4 and Table 4, illustrating the sensitivity and specificity of each parameter for predicting malignancy.

### 3.2. Multidimensional Data Analysis

Given that no single parameter could effectively classify all cases, a machine learning model was employed to improve classification accuracy by considering the interactions between multiple attributes. The analysis used a support vector machine (SVM) classifier, with the sequential floating-forward search (SFFS) algorithm to select the most relevant parameters. The detailed methodology is described in the material and methods section. This approach identified a subset of eight parameters (including SAI, amplitude asymmetry, and phase difference parameters) that significantly improved the classification accuracy.

For the machine learning analysis, the bootstrapping method was applied to the full training dataset, to mimic the workflow applied in the univariate analysis. To follow the established principles of model evaluation in machine learning, it is the cross-validation experiment that should be considered as a more reliable assessment of SVM-associated empirical risk.

#### 3.2.1. Classification to 3 Classes

**Figure 5:**
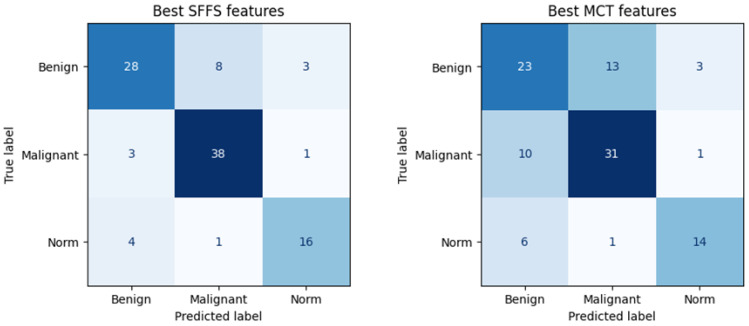
Confusion matrix obtained for subsets of significant features determined using the SFFS algorithm and in the MCT univariate analysis. The matrix represents correctly (on the diagonal) and incorrectly (off the diagonal) classified cases. Classifier testing was performed in the 5-fold cross-validation mode.

The SFFS-based model showed superior performance compared to the model trained using parameters selected in MCT analysis, correctly classifying 72% of benign lesions, 91% of malignant lesions, and 76% of normophonic subjects. While for MCT it was respectively correctly classified 59% benign lesions, 74% malignant lesions and 67% of normophonic subjects. The results of the SFFS and MCT analyses are presented in the confusion matrices in Figure 5. These findings highlight the effectiveness of integrating multiple attributes within a machine learning framework, which exceeds the predictive performance of individual parameters whose significance was established independently through univariate statistical analysis.

#### 3.2.2. Multidimensional Data Analysis–Classification to 2 Classes: Any Organic Lesion–Norm

The SVM classifier determines the probability of membership in each category for every case being classified. The decision to assign a patient to a specific category is made based on the highest probability. Since the classification outcome, which is the probability of belonging to a selected category, is a continuous value, it is possible to assess the quality of the classification by performing ROC analysis along with the calculation of the standard evaluation metrics, such as balanced accuracy, sensitivity and specificity. In contrast, however, to threshold classifiers built in statistical analysis, the latter two are calculated based on the multidimensional decision hypersurface instead of single cut-off points.

**Figure 6:**
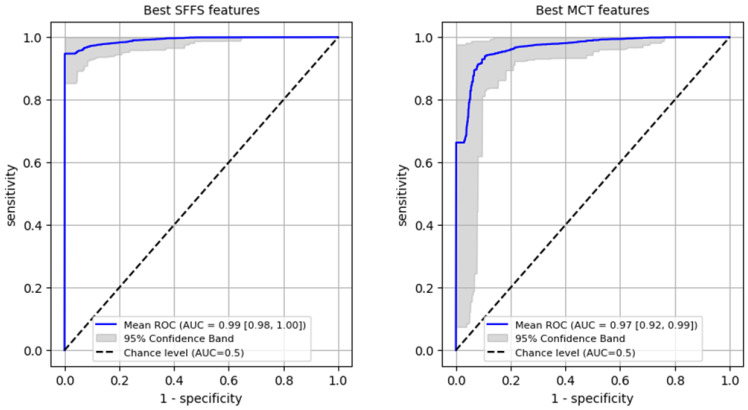
ROC curves training set for differentiating normophonic subjects from patients with any organic lesion of the glottis (both benign and malignant).

Firstly, a ROC analysis was conducted on the training set to differentiate normophonic subjects from patients with any organic lesion of the glottis, achieving an AUC of 0.99 (95% CI: 0.98–1.0) for the SFFS algorithm and an AUC of 0.97 (95% CI: 0.92–0.99) for the MCT analysis (Figure 6). In case of the SFFS-selected parameters and with lesion class set as a positive label, the model yielded sensitivity of 96% (95% CI: 0.91–1.0), specificity = 99% (95% CI: 0.94–1.0). For MCT-based analysis similar values o sensistivity of 93% (95% CI: 0.84–0.99), specificity = 97% (95% CI: 0.9–1.0)

**Figure 7:**
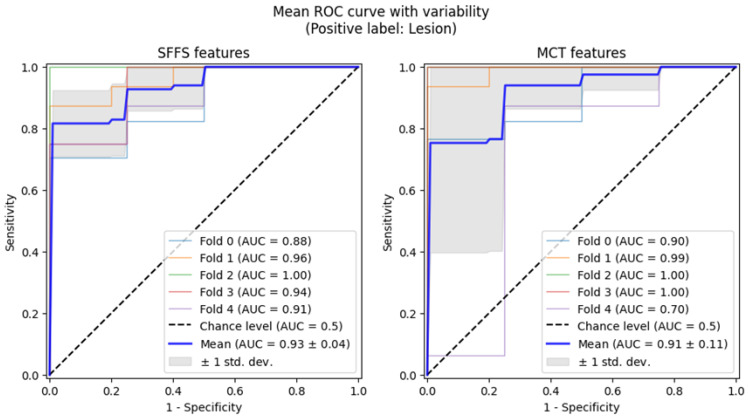
ROC curves testing with cross-validation for differentiating normophonic subjects from patients with any organic lesion of the glottis (both benign and malignant).

Subsequently, 5-fold cross-validation testing was conducted to differentiate normophonic subjects from patients with any organic lesion of the glottis, whether benign or malignant. This testing achieved a mean AUC of 0.93 for the SFFS algorithm and a mean AUC of 0.91 for the MCT univariate analysis (Figure 7). The sensitivity metrics remained relatively high, at 97.5% for the SFFS analysis and 93.8% for the MCT analysis. However, specificity decreased to 62% for SFFS and 71% for MCT.

#### 3.2.3. Multidimensional Data Analysis–Classification to 2 Classes: Malignant Lesion–Benign Lesion

**Figure 8:**
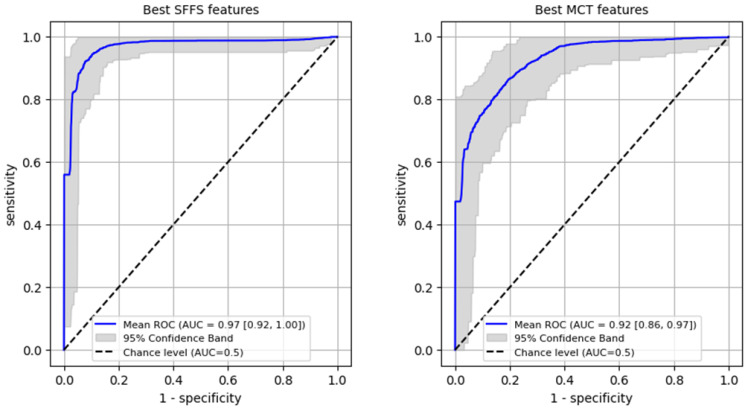
ROC curves training set for differentiating benign from malignant lesions of the glottis.

ROC analysis for differentiating benign from malignant lesions of the glottis on the training set was performed reaching AUC 0.97 (95% CI: 0.92–1.0) for SFFS algorithm and AUC 0.92 (95% CI: 0.86–0.97) for MCT univariate analysis (Figure 8). In case of the SFFS-selected parameters and with malignancy class set as a positive label, the model yields sensitivity of 95% (95% CI: 0.85–1.0), specificity = 94% (95% CI: 0.86–1.0). For MCT-selected attributes, sensitivity = 87% (95% CI: 0.74–1.0), specificity = 87% (95% CI: 0.69–1.0).

**Figure 9:**
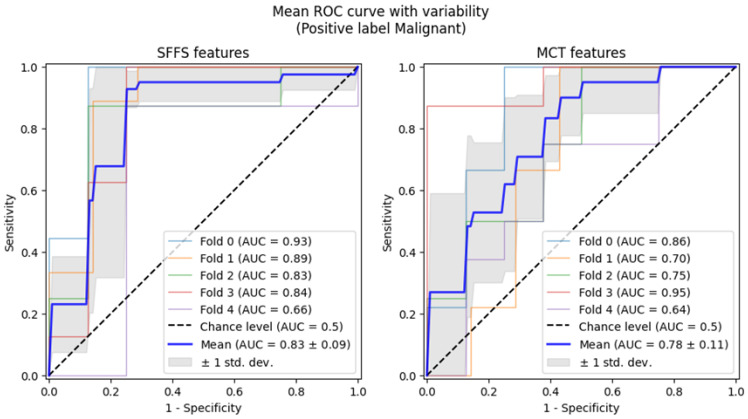
ROC curves testing with cross-validation for differentiating benign from malignant lesions of the glottis.

Subsequently, 5-fold cross-validation testing was conducted to differentiate benign from malignant lesions of the glottis. This testing achieved a mean AUC of 0.83 for the SFFS algorithm and a mean AUC of 0.78 for the MCT univariate analysis (Figure 9). Only the specificity for SFFS remains relatively high = 90.6% while the sensitivity = 79.3% for the SFFS and both sensitivity = 66.8%, and specificity = 74.4% for MCT univariate analysis decreased.

Summarizing, analysis using cross-validation demonstrated that the SVM classifier trained on the SFFS-selected features achieved an AUC of 0.93 in differentiating normophonic subjects from patients with organic lesions and AUC of 0.83 for distinguishing benign from malignant lesions. This underscores the potential of machine learning in enhancing diagnostic accuracy, particularly in the early detection of glottic cancer.

## 4. Discussion

The study shows that Stiffness Asymmetry Index (SAI) is a valuable parameter for detecting organic lesions and predicting malignancy in the vocal folds but it cannot definitively distinguish benign from malignant lesions alone. However, applying the advanced machine learning models, which integrate multiple parameters showed to be promising approach for improving diagnostic accuracy. The findings suggest that SAI, while not sufficient on its own, plays a key role when combined with other dynamic measures of vocal fold function in distinguishing between benign and malignant lesions.

In our study, we assessed vocal fold stiffness as a means of predicting glottic malignancy, using a specially designed Stiffness Asymmetry Index (SAI) derived from the laryngotopographic (LTG) analysis using the High-speed recordings. This index was developed to non-invasively describe the changes in vocal fold structure caused by organic lesions. The results of our study underscore the significant role that vocal fold pliability plays in the diagnosis of glottic lesions, especially when distinguishing between benign and malignant tumors. Growth of the lesion alters the pliability of the vocal fold and it becomes “stiffer” as its ability to vibrate during phonation is reduced. One of the main parts of the vocal fold participating in the vibrations is the membranous portion covering the muscle layer. The wave-like motion of this structure is called mucosal wave and it is only visible during the functional endoscopic examination, such as LVS and HSV. While LVS is commonly used to assess vocal fold vibrations, it fails in cases of aperiodic vibrations, such as those seen in patients with severe dysphonia presented often by patients with glottic cancer. HSV, in contrast, provides a more effective method by recording real-time oscillations thus overcoming the limitations of LVS in such cases [18,19].

Mucosal wave is usually a main part of the subjective assessment of vocal fold vibrations using the HSV along with the glottal closure, symmetry, periodicity and amplitude [20]. These characteristics are commonly assessed subjectively by the clinician as part of glottal function examination, mostly by the LVS. However, a more effective and objective assessment could help clinicians differentiate between the causes of dysphonia [21]. While the benign lesions are usually soft resulting in slight altering of the involved vocal fold’s elasticity and pliability, cancerous lesions present different characteristics due to the infiltration of deeper structures. In the case of glottal cancer, studies report the absence of vibratory amplitude in the affected region which could be visualized as a non-vibrating area in LTG. To fill the gap for the objective evaluation of vocal fold pliability which is a crucial aspect in the glottic lesions assessment, we proposed the Stiffness Asymmetry Index (SAI). The parameter describes the stiffness of the affected vocal fold in comparison to non-affected vocal fold based on the LTG maps using the HSV recordings. Due to the high sampling rate, changes in the oscillations are assessed based on the real vocal fold movements allowing the precise evaluation of involved by the lesion areas and their function. Moreover, HSV is successful in the aperiodic vibrations which are often presented in the severe dysphonia caused by the increased mass of vocal folds [22,23].

In our study, the SAI emerged as one of the most promising parameters. It showed excellent performance in detecting any organic lesion and predicting malignancy, both in terms of the area under the ROC curve (AUC) and its sensitivity and specificity (respectively ROC AUC 0.91, 0.8, sensitivity 0.832, 0.733 and specificity 0.903, 0.812). These findings support previous studies that have demonstrated the importance of objective measures in detecting glottic malignancy [12,18]. While benign lesions typically exhibit soft tissue characteristics that result in only slight alterations to vocal fold elasticity, malignant lesions present with more substantial changes due to the infiltration of deeper structures, leading to a reduced mucosal wave and increasing the stiffness of vocal fold. Those changes resulting in the absence of vibratory amplitude in the affected region could be visualized as a non-vibrating area in LTG allows for more accurate discrimination between benign and malignant lesions [18,19].

Besides SAI, the study showed significance of the parameters related to phase difference and amplitude asymmetry of vocal folds which were also significant in our previous study, which focused solely on kymographic parameters for detecting malignancy [24]. These consistent findings highlight the importance of amplitude asymmetry and phase difference in the non-invasive assessment of glottic lesions. In that earlier work, we also evaluated a parameter that specifically measures the amplitude of the vocal fold affected by the lesion. Given the importance of vocal fold pliability and the recommendation by some authors to assess each vocal fold separately, we decided to include this parameter in our analysis [25,26]. This approach indirectly evaluates tissue stiffness by considering the asymmetry between the healthy and affected vocal folds. Average amplitude of affected vocal fold was shown to be a significant predictor of vocal fold malignancy, alongside other kymographic parameters. However, the ROC AUC for this parameter was slightly lower than that obtained for the SAI in this study (0.7 vs. 0.8) [24].

Also in favor of evaluating the two vocal folds separately is the fact that vocal fold stiffness depends on age and gender. It is stated that stiffness of vocal folds is higher in women, and also regardless of gender is associated with aging [8]. By comparing the affected vocal fold to the healthy fold, our approach allows for a more accurate assessment of the lesion’s impact on vocal fold function, taking into account these individual differences. Assessment of the tissue stiffness was performed by the LTG to detect periodic and non-periodic vocal fold vibrations in HSV recordings. The method enables quantitative evaluation of the spatial characteristics of the amplitude and phase of phonatory oscillations of the vocal folds due to the Fourier transformation of brightness for each pixel across a sequence of recorded HSV images. Obtained LTG maps present spectral parameters e.g., fundamental frequency and amplitude with assigned pseudocolors for the simple qualitative interpretation. Moreover, LTG visualizes the non-vibrating areas due to the lack of mucosal wave and vibration amplitude which was a base for the creation of the SAI.

Despite the significant differences in the median values of SAI between the groups of patients (norm, benign, malignant) they could not be clearly distinguished based only on this parameter. The median values of SAI were respectively about 0.6 for the malignant lesions, 0.3 for benign lesions, and 0.06 for the normophonic subjects. However, there are cases with very low values of SAI more suitable for the normophonic or benign lesion group that were revealed to be cancerous lesions. Exploring those cases in detail, it appeared they are small superficial lesions, sometimes located in the posterior part of the glottis. Therefore, they were having only a slight influence on the vocal fold oscillations. On the other hand, there were high values of SAI noted for the lesions diagnosed as benign. According to the detailed assessment of the lesions’ characteristics, we assumed that it could be a result of hemorrhagic or fibrotic polyps. Those lesions are higher in mass and alter the pliability of the vocal fold more than the jellylike mass of typical polypoid lesions. Moreover, the differences could be caused by the size of the lesions because a big mass could affect the vibrations of healthy vocal fold during the phonatory movements.

Effort is being made to non-invasively classified the glottis lesions before the histopathological examination, which remain the gold standard. The meta-analysis showed that while LVS identifies almost all cases with cancer, only 2/3 of patients with non-invasive lesions are identified as non-cancerous [27]. According to the literature, the best results for the preoperative assessment give the combination of different methods for the endoscopic assessment e.g., functional and with the visualization of the vasculature. Therefore, the authors indicated the need for non-invasive and objective assessment before the surgery [28]. Study performed by Volgger et al. showed that combination of HSV and NBI reach high sensitivity (100%) and specificity (79.4%) in the differentiation between benign/premalignant and malignant lesions [29]. Machine learning techniques, are also being applied to obtain better results, such as the support vector machine (SVM) classifier used in our study, proved to be effective in handling the complex interactions between the parameters characterizing the vocal fold oscillations. The results from the multidimensional data analysis, which integrated multiple parameters including SAI, showed a significant improvement over the univariate analysis. This approach demonstrated better classification accuracy, with the SVM model achieving an AUC of 0.93 for detecting organic lesions (vs. 0.92 for univariate analysis) and 0.83 for distinguishing malignant from benign lesions (vs. 0.79 for univariate analysis). These findings emphasize the potential of machine learning models in clinical applications, where they can enhance diagnostic accuracy by considering the interplay between various parameters. Machine learning methods are now widely implemented in the diagnostic process mainly using the white light and NBI endoscopic images of the larynx to improve the diagnostic process and detect the malignant lesions at the early stage. Zhao et al. investigate different ML models including random forest (RF), support vector machine (SVM), and decision tree (DT), to identify risk factors and predict the malignant lesions using the NBI in a group of 200 patients. The study showed that RF-based model had the best results which were: the accuracy—0.96, precision—0.90, recall 1.00 and F1 index—0.95, and AUC value 0.97. While for the SVM the results were slightly worse than for the RF model: the accuracy—0.91, precision—0.86, recall 0.95 and F1 index—0.90, and AUC value 0.95 but better than in our SVM-based model using the SAI and kymographic parameters based on the HSV [30]. Chinese group evaluate six classical deep learning techniques for the classification of the vocal fold leukoplakia showing the best performance for the GoogLeNet, ResNet, and DenseNe in which the highest overall accuracy of white light image classification is 0.9583, while the highest overall accuracy of NBI image classification is 0.9478 [31]. Other studies proposed also self-made deep-learning algorithms to detect and classify the lesions during the real-time endoscopy obtaining sensitivity for the carcinoma classification between 71% and 78% and for benign vocal cord lesions with a sensitivity between 70% and 82%. That approach could support the clinical decision making during the appointments and it not time consuming like other methods requiring further analysis [32]. The meta-analysis from the 2020 showed that the SVM is the most common algorithm for the detection of voice disorders. [33] However, there are many other well established and self-developed machine learning techniques that give various results for the classification of glottic lesions based on different endoscopic methods.

Besides the use of only one machine learning methods which is SVM in this study, there are several other limitations that should be addressed in future research. First, we included only patients with unilateral lesions, which allowed for direct comparisons between the affected and healthy vocal fold in each patient. While this methodology is robust, it does not fully account for bilateral lesions, which are common in clinical practice.

Besides that, the limitation is the time-consuming nature of the methodology, along with the need for specialized training in the use of HSV and SAI, may limit its widespread adoption in routine clinical settings. Further improvements in the speed and ease of the process, as well as the development of user-friendly software, will be crucial for the implementation of this technique in daily practice [23].

We did not provide the threshold cutoff-values because of the implemented methodology. As we investigated only a group of 102 patients after all exclusions, we had performed a ROC analysis with boostrapping to compensate small sample size. Youden indexes are calculated for the mean ROC curves, but the threshold cutoff-values can only be given for the full original data. Otherwise, determination of a cutoff value is ambiguous.

## 5. Conclusions

In conclusion, Stiffness Asymmetry Index (SAI) is a promising parameter for the non-invasive distinguishing between benign and malignant glottic lesions. Applied to the kymographic analysis using the high-speed videoendoscopy (HSV) By utilizing a support vector machine (SVM) classifier it achieved an AUC of 0.93 for identifying organic lesions and 0.83 for differentiating malignancies from benign lesions, outperforming traditional univariate threshold-based analyses. While no single parameter based on high-speed videoendoscopy (HSV) can definitively predict early glottic cancer, machine learning models which integrate multiple parameters, can further enhance diagnostic performance. The results of this study suggest that SAI, in combination with other kymographic parameters derived from the HSV, could serve as an important adjunct to histopathological evaluation, offering a non-invasive method for early detection and improved clinical decision-making.

## Figures and Tables

**Table 1:** Kymographic parameters included in the study and their descriptions. %FL—percent of a vocal fold length.

	Parameter	Description		Parameter	Description
1	AmpMaxInd (%FL)	Index of maximum glottal gap amplitude	20	OQAvg_3/3 (%)	Open Quotient 1/3 anterior part of the glottis
2	AmpRMaxInd (%FL)	Index of maximum right vocal fold edge movement amplitude	21	AmplAsymAvg (%)	Average amplitude asymmetry
3	AmpLMaxInd (%FL)	Index of maximum left vocal fold edge movement amplitude	22	AmplAsymAvg_1/3 (%)	Average amplitude asymmetry 1/3 posterior part of the glottis
4	AmpCenter (%FL)	Index of net glottal gap amplitude	23	AmplAsymAvg_2/3 (%)	Average amplitude asymmetry 1/3 middle part of the glottis
5	AmpRCenter (%FL)	Index of net right vocal fold edge movement amplitude	24	AmplAsymAvg_3/3 (%)	Average amplitude asymmetry 1/3 anterior part of the glottis
6	AmpLCenter (%FL)	Index of net left vocal fold edge movement amplitude	25	AmplAsymWeighted (%)	Weighted amplitude asymmetry
7	AmpAvg (%FL)	Average glottal gap amplitude	26	PhaseAsymAvg (%)	Average phase asymmetry
8	AmpAvg_1/3 (%FL)	Glottal gap amplitude 1/3 posterior part of the glottis	27	PhaseAsymAvg_1/3 (%)	Average phase asymmetry 1/3 posterior part of the glottis
9	AmpAvg_2/3 (%FL)	Glottal gap amplitude 1/3 middle part of the glottis	28	PhaseAsymAvg_2/3 (%)	Average phase asymmetry 1/3 middle part of the glottis
10	AmpAvg_3/3 (%FL)	Glottal gap amplitude 1/3 anterior part of the glottis	29	PhaseAsymAvg_3/3 (%)	Average phase asymmetry 1/3 anterior part of the glottis
11	AmpLAvg (%FL)	Average left vocal fold amplitude	30	PhaseAsymWeighted (%)	Weighted Average phase asymmetry
12	AmpRAvg (%FL)	Average right vocal fold amplitude	31	Rel2CommonAmplAvg (%)	Average relative to common amplitude ratio
13	Nonclosing (%FL)	Non-closing part of vocal folds	32	PhaseDiffAvg (°)	Average phase difference
14	Nonopening (%FL)	Non-opening part of vocal folds	33	PhaseDiffAvg_1/3 (°)	Average phase difference 1/3 posterior part of the glottis
15	EffectiveArea (%)	Glottal effective area	34	PhaseDiffAvg_2/3 (°)	Average phase difference 1/3 middle part of the glottis
16	RGGA (%)	Relative Glottal Gap Area	35	PhaseDiffAvg_3/3 (°)	Average phase difference 1/3 anterior part of the glottis
17	OQAvg (%)	Average Open Quotient	36	PhaseDiffWeighted (°)	Weighted phase difference
18	OQAvg_1/3 (%)	Open Quotient 1/3 posterior part of the glottis	37	AbsPhaseDiffAvg (°)	Average absolute phase difference
19	OQAvg_2/3 (%)	Open Quotient 1/3 middle part of the glottis	38	AbsPhaseDiffWeighted (°)	Weighted absolute phase difference

**Table 2:** Parameters which differ simultaneously between the normophonic patients, subjects with benign and malignant lesions (U Mann-Whitney test). Statistic value presents the difference level between investigated groups—the higher is the value of statistic the higher is the measured difference.

Parameter	Groups	*p*-Value	Statistic
AbsPhaseDiffAvg	Benign vs. Malignant	0.0096	544.5
	Norm vs. Benign	0.0002	169.0
	Norm vs. Malignant	<0.0001	132.5
AbsPhaseDiffWeighted	Benign vs. Malignant	0.0097	545.0
	Norm vs. Benign	0.0004	178.5
	Norm vs. Malignant	<0.0001	143.0
AmplAsymAvg	Benign vs. Malignant	0.0001	416.0
	Norm vs. Benign	<0.0001	124.5
	Norm vs. Malignant	<0.0001	62.0
AmplAsymAvg_2/3	Benign vs. Malignant	0.0010	505.0
	Norm vs. Benign	0.0001	160.0
	Norm vs. Malignant	< 0.0001	112.5
AmplAsymWeighted	Benign vs. Malignant	0.0001	412.0
	Norm vs. Benign	<0.0001	93.0
	Norm vs. Malignant	<0.0001	47.0
SAI	Benign vs. Malignant	<0.0001	337.5
	Norm vs. Benign	<0.0001	123.5
	Norm vs. Malignant	<0.0001	34.0

**Table 3:** Parameters highlighted in the multiple comparison analysis for which ROC analysis with bootstrapping was performed (differentiation of normophonic subjects from patients with any organic lesion of the glottis). The table contains the Youden index values and the corresponding sensitivity and specificity values with 95% confidence intervals. Sensitivity (True Positive Rate): Measures the proportion of actual positive cases correctly identified (i.e., how well the model detects malignant lesions); Specificity (True Negative Rate): Measures the proportion of actual negative cases correctly classified (i.e., how well the model avoids false positives in detecting malignancy).

	Parameter	Youden’s Index (95% CI)	Sensitivity (95% CI)	Specificity (95% CI)
1	SAI	0.735 (0.612–0.882)	0.832 (0.623–0.966)	0.903 (0.737–1.0)
2	AmplAsymAvg	0.715 (0.581–0.833)	0.777 (0.662–0.903)	0.938 (0.786–1.0)
3	AmplAsymAvg_2/3	0.633 (0.482–0.788)	0.764 (0.5–0.911)	0.869 (0.667–1.0)
4	AmplAsymWeighted	0.595 (0.395–0.779)	0.827 (0.697–0.952)	0.921 (0.765–1.0)
5	AbsPhaseDiffAvg	0.627 (0.435–0.8)	0.808 (0.587–0.919)	0.819 (0.636–1.0)
6	AbsPhaseDiffWeighted	0.595 (0.395–0.779)	0.783 (0.625–0.938)	0.812 (0.571–1.0)

**Table 4:** Parameters highlighted in the multiple comparison analysis for which ROC analysis was performed (differentiating benign from malignant lesions of the glottis). The table contains the Youden index values, sensitivity and specificity values with 95% confidence intervals.

	Parameter	Youden’s Index (95% CI)	Sensitivity (95% CI)	Specificity (95% CI)
1	SAI	0.545 (0.379–0.705)	0.733 (0.54–0.913)	0.812 (0.621–0.955)
2	AmplAsymAvg	0.538 (0.0.362–0.705)	0.763 (0.538–0.889)	0.775 (0.634–0.941)
3	AmplAsymAvg_2/3	0.440 (0.259–0.606)	0.624 (0.375–0.825)	0.816 (0.629–0.974)
4	AmplAsymWeighted	0.503 (0.334–0.667)	0.725 (0.442–0.895)	0.778 (0.605–0.976)
5	AbsPhaseDiffAvg	0.366 (0.2–0.532)	0.668 (0.341–0.923)	0.698 (0.424–0.956)
6	AbsPhaseDiffWeighted	0.369 (0.189–0.544)	0.648 (0.355–0.895)	0.72 (0.432–0.968)

## Data Availability

The data presented in this study are available on request from the corresponding author.

## Supplementary Materials

The following supporting information can be downloaded at: https://www.mdpi.com/article/10.3390/cancers17081376/s1, Supplementary S1—short-term kymographic analysis.

## Abbreviations

The following abbreviations are used in this manuscript:

SAI	Stiffness Asymmetry Index
HSV	High-Speed Videoendoscopy
TPR	True Positive Rate
FPR	False Positive Rate
ROC	Receiver Operating Characteristic
AUC	Area Under The Curve
SVM	Support Vector Machines
SFFS	Sequential Floating-Forward Search
MCT	Multiple Comparisons Test
	Abbreviations for parameters with description in Table 1
