# Supplementary material for: High-Speed Videoendoscopy and Stiffness Mapping for AI-Assisted Glottic Lesion Differentiation
Source: Cancers (Basel). 2025 Apr 21;17(8):1376. doi: 10.3390/cancers17081376 (PMC12025992; doi:10.3390/cancers17081376)
Supplement: Supplementary file 1 [file cancers-17-01376-s001.zip › cancers-3503208-supplementary.pdf]

Kymography - Short-term kymo analysis

Author: MSc Anna Racino, Ph.D. Marcin Just, Ph.D. Michał Tyc (DiagNova Technologies)
Date: 2021.05.01

AliCam (from DiagNova Technologies Sp. z o.o. **Advances Larynx Imager System** kit) is required for high speed recording. There is a foot switch dedicated to control the camera **AliCam** and the light from illuminator **AliLum**.

Another kymographic analysis - *Short-time kymo analysis* (Fig. 10) begins on the *Kymographic sections* tab. The tab on the left side has an image showing the cropped vocal folds with a slider to select the location of the cross-section 12. In order to select the cross-section of the folds of interest, set the slider in the appropriate place by moving it up or down with the cursor. As the slider is moved, the kymographic picture corresponding to a given cross-section appears on the right.

Likewise, the frame representing the vocal folds corresponding to a given instant in time may be changed. For this purpose, the moment in which the frame was recorded should be determined by moving the slider on the kymographic section 13, which automatically allows setting the appropriate cropped frame.

Below the kymographic section, there are buttons that allow you to save images to the report. The *Add joint kymographic section* button allows saving an image showing a cropped frame with vocal folds along with the appropriate kymographic section 14. The *Add set of sections* button allows adding an image that represents the cropped frame along with the kymography of the vocal folds in seven places of the cross-sections 15 (selectable places on the list next to it).

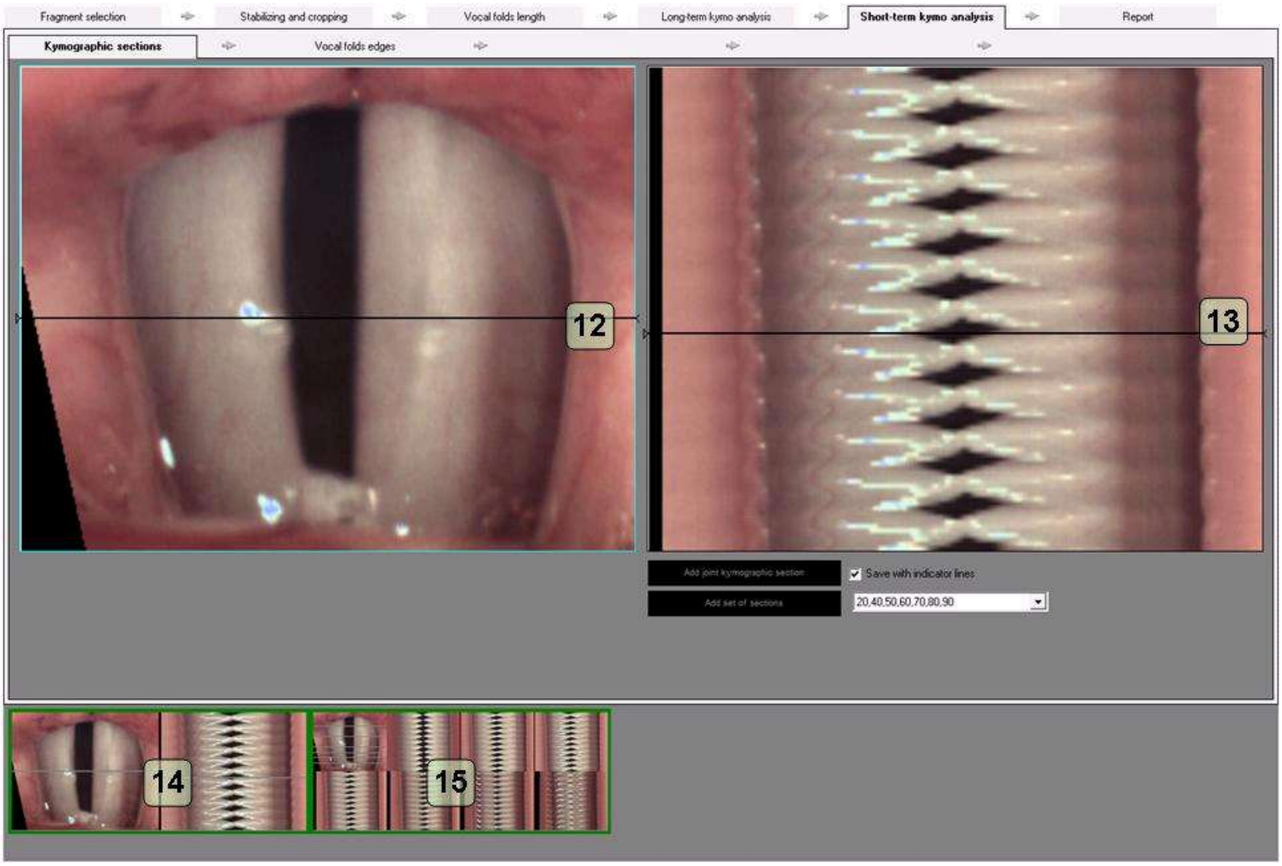

Fig. 10. Kymographic analysis window – Short-term kymo analysis -> Vocal folds edges

The next tab is *Vocal folds edges* (Fig. 11). On the image of the cropped vocal folds shows there are two green sliders, located halfway up the image. The sliders allow defining the area to be considered in kymographic analysis. The designated width allows correcting the degree of visible opening of the vocal folds.

Below the image of the cropped vocal folds there is a button *Optimize detection parameters* 16. After positioning the image of the vocal folds at the moment of their opening, pressing the button 16 will automatically adjust a set of five pairs of points to the right and left edges of the vocal folds. In the case of not all points match correctly, their position can be corrected "manually" by moving individual points to the right or left by moving using mouse. After the points have been set in the vocal fold image, the user may proceed to determining the vocal folds opening area in the kymographic image.

In the kymographic section, the program paints blue the areas where the folds are open. After pressing the button below the kymographic image *Optimize detection parameters* 17, the program finds areas where the folds are open. In the event that not all areas fit properly, their positioning can be corrected "manually" by moving single points 18 to the right or left, reducing or increasing the brightness range, which the program takes into account when determining the area of folds in the kymographic image. It is important for the user to pay attention to which interval between the points is incorrectly determined, since the correction is performed using the point closest to this section. By moving the slider to the left and right, it is possible to reduce the brightness range (decrease the selected area) and increase the brightness range (increase the selected area).

For the correct determination of parameters and phonovibrograms, the number of complete cycles (full opening and closing) of the folds function occurring on the analyzed recording should also be indicated (*Number of full cycles*).

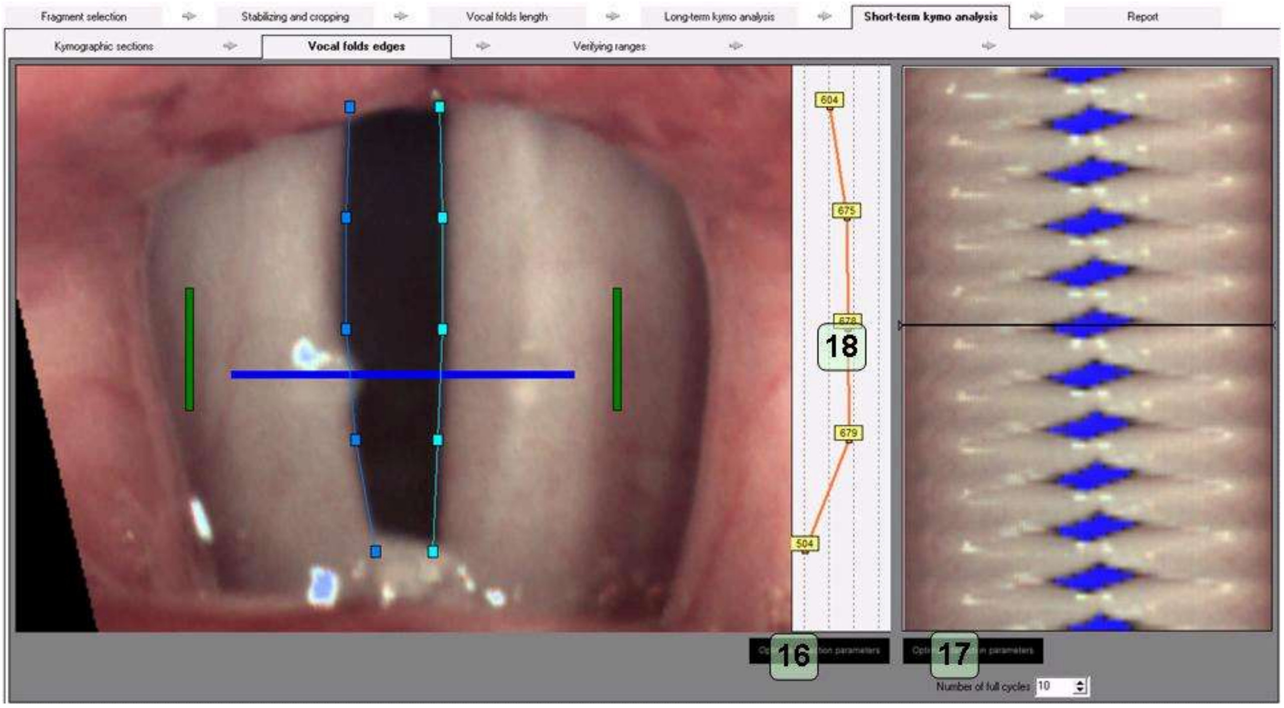

Fig. 11. Kymographic analysis window – Short-term kymo analysis -> Vocal folds edges

In the *Verifying ranges* tab (Fig. 12) there is a kymographic image and an image of the vocal folds. The simplified phonovibrograms graph 19 allows for precise verification of the marked ranges of vocal folds lengths and vocal folds opening. Additionally, in the tab there are graphs showing the edges of the vocal folds. The graph 20 shows the edges of the folds with symmetry correction (the *Calculate symmetry correction* button). In the graph 21 the edges of the vocal folds are shown according to the settings made on the *Vocal folds edges* tab. In this tab, the user can also correct the number of complete cycles (*Declared number of cycles*).

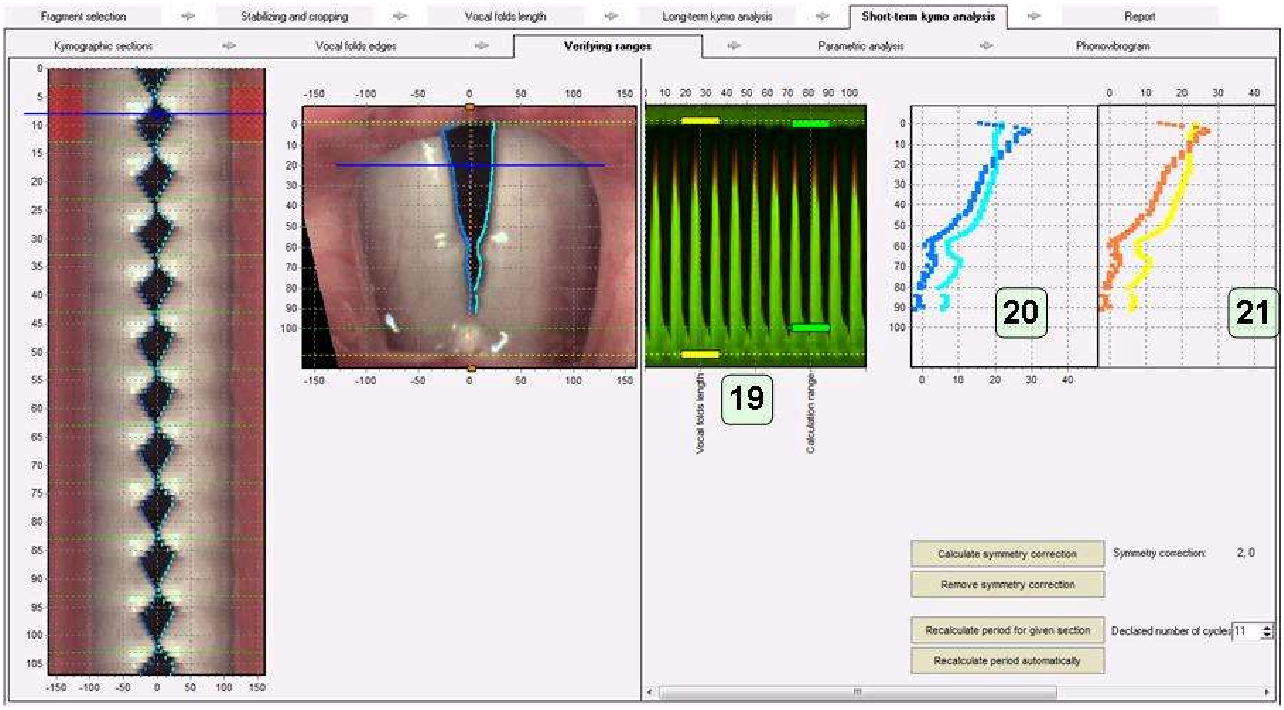

Fig. 12. Kymographic analysis window – Short-term kymo analysis -> Verifying ranges

Additionally, the user can select Phase difference view (Fig. 13) and open a new chart.

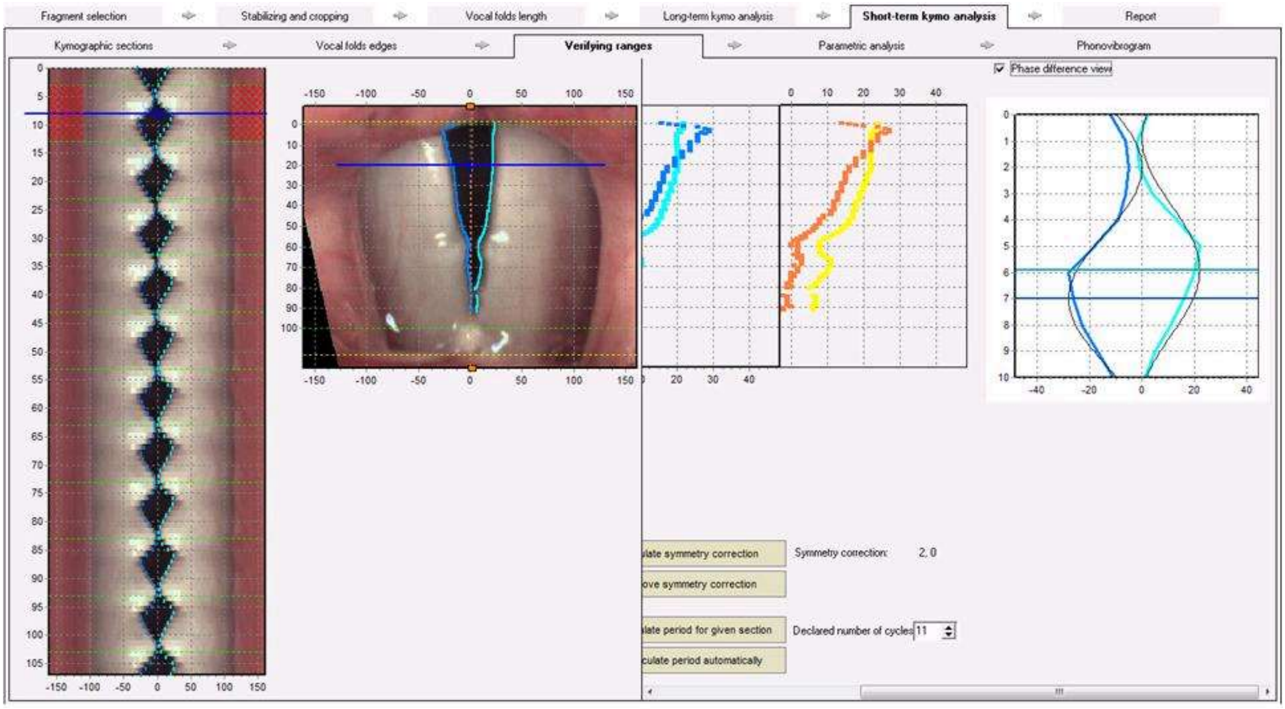

Fig. 13. Kymographic analysis window – Short-term kymo analysis -> Verifying ranges; Phase difference view

After making the appropriate corrections, you can go to the next tab - *Parametric analysis* (Fig. 14). This tab displays graphs and short-term parameters determined on the basis of the settings.

Charts can be added to the list of saved images 22 for the report by double-clicking on the chart.

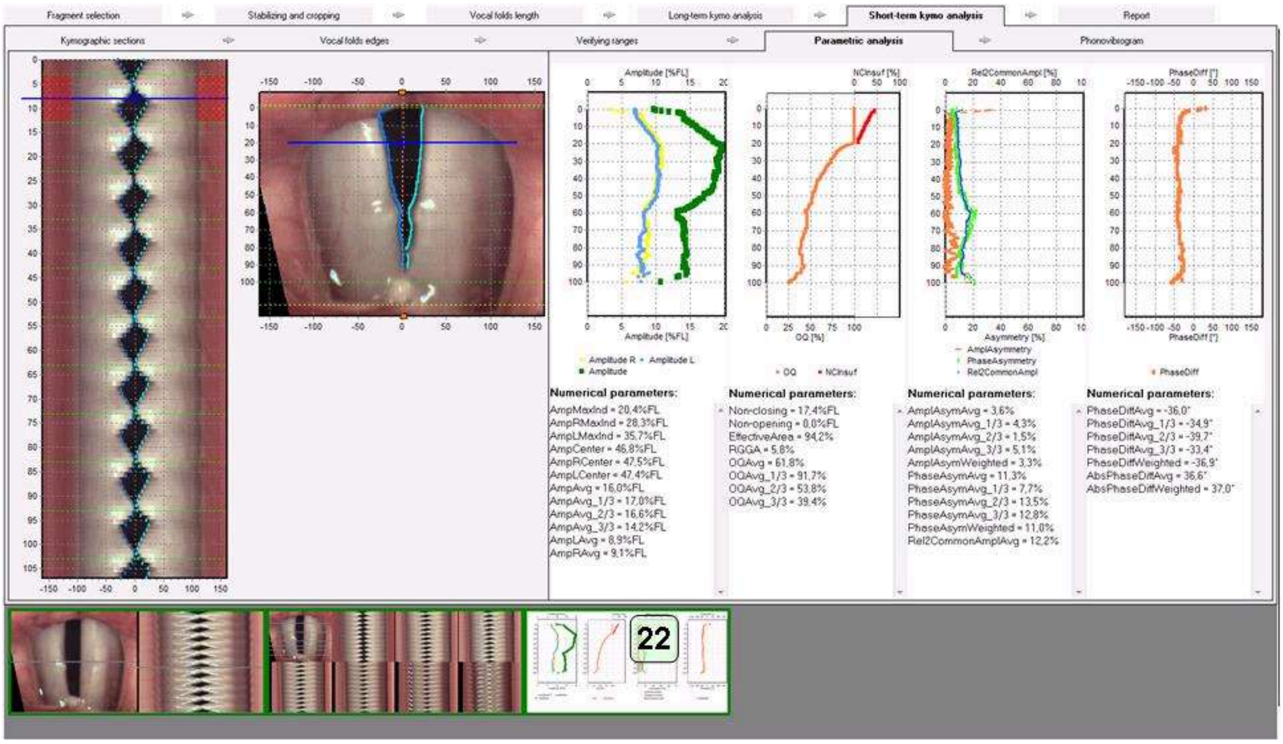

Fig. 14.Kymographic analysis window – Short-term kymo analysis -> Parametric analysis

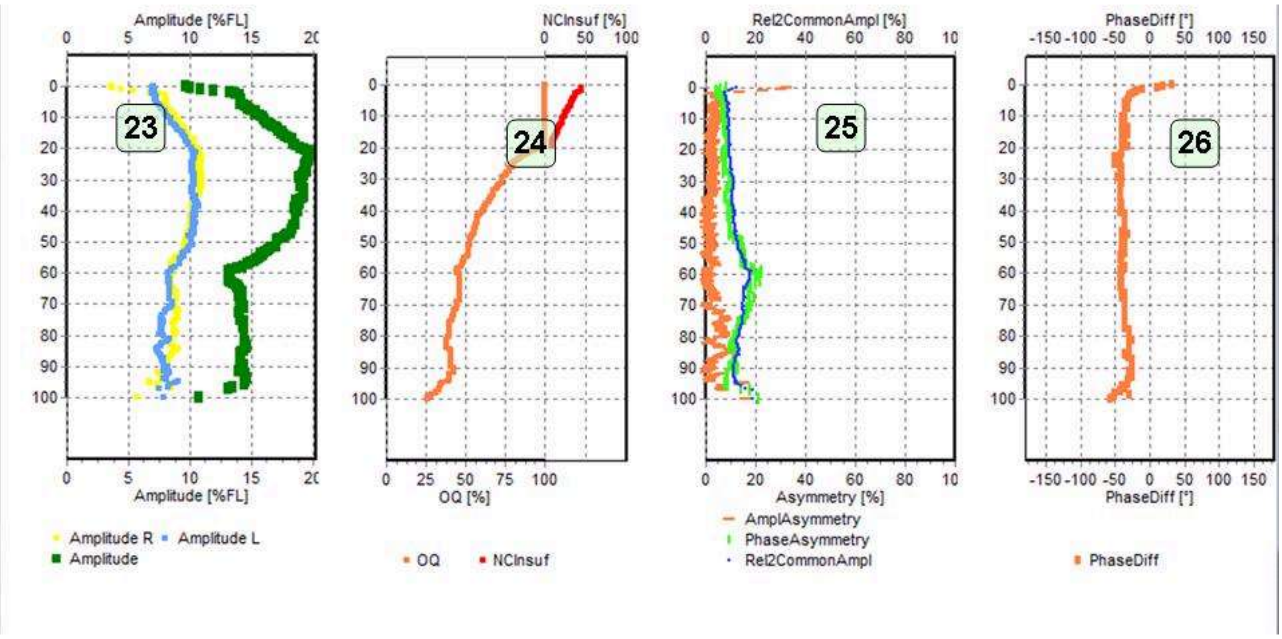

Fig. 15. Kymographic analysis window – Short-term kymo analysis -> Parametric analysis

Description of short-term parameters (Fig. 15) is presented in the tables below. Amplitude analyzer examines the movements of the vocal folds perpendicular to their axis. The range of changes in the position of the left and right edges of the fold is determined. The analyzer is associated with one graph 23 with three data series (description in table 2) and global parameters presented in table 3. The unit of the X axis is the percentage of the fold length (% FL).

Table 2 Data series in an amplitude analyzer graph:

PARAMETER	DESCRIPTION
Amplitude	**Glottal gap amplitude** The resultant amplitude of the movement of the folds (glottis gap width) for a given point on their axis. The Y axis specifies the position on the fold axis.

PARAMETER	DESCRIPTION
Amplitude R	**Right vocal fold edge movement amplitude** The resultant amplitude of the movement of the right fold for a given point on the fold axis. The Y axis specifies the position on the fold axis.
Amplitude L	**Left vocal fold edge movement amplitude** The resultant amplitude of the movement of the left fold for a given point on the fold axis. The Y axis specifies the position on the fold axis.

Table 3 Global parameters of the amplitude analyzer:

PARAMETER	DESCRIPTION
AmpMaxInd (%FL)	**Index of maximum glottal gap amplitude** The indicator of the greatest resultant amplitude of the folds movement. Specifies the location along the fold axis where there is the greatest difference between the distance between the fold edges when fully open and fully closed. The result is based on the total length of the fold (for example, a value of 50% means that the maximum amplitude is in the half of the fold length).
AmpRMaxInd (%FL)	**Index of maximum right vocal fold edge movement amplitude** The indicator of the greatest amplitude of the movement of the right fold. Specifies the location on the fold axis where there is the greatest differ-ence between the edge of the right fold when fully open and fully closed. The result is based on the total length of the fold (for example, a value of 50% means that the maximum amplitude is in the half of the fold length).
AmpLMaxInd (%FL)	**Index of maximum left vocal fold edge movement amplitude** The indicator of the greatest amplitude of the movement of the left fold. Specifies the position on the fold axis where there is the greatest difference between the left fold edge when fully open and fully closed. The result is based on the total length of the fold (for example, a value of 50% means that the maximum amplitude is in the half of the fold length).
AmpCenter (%FL)	**Index of net glottal gap amplitude** Center of gravity of the resultant amplitude of the folds movement. The result is relative to the total length of the fold (for example, a value of 50% means that the amplitude center of gravity is half the fold length).
AmpRCenter (%FL)	**Index of net right vocal fold edge movement amplitude** The center of gravity of the amplitude of the movement of the right fold. The result is based on the total length of the fold (for example, a value of 50% means that the amplitude center of gravity is half the length of the fold).
AmpLCenter (%FL)	**Index of net left vocal fold edge movement amplitude** The center of gravity of the amplitude of the movement of the left fold. The result is relative to the total length of the fold (for example, a value of 50% means that the amplitude center of gravity is half the fold length).
AmpAvg (%FL)	**Average glottal gap amplitude** Average resultant amplitude of folds movement for folds. Averaged values for all points along their length and for their back 1/3 length, middle 1/3 length and front 1/3 length respectively.

The fold tactility analyzer examines the movement of the vocal folds perpendicular to their axis. The percentage of the time that the folds cycle when the folds are fully closed (edges touching) is determined. The analyzer is associated with one graph 24 with two data series (description in table 6.4) and global

parameters presented in table 6.5. The unit of the X axis is the opening factor.

Table 4 Data series in the plot of the fold closure analyzer:

PARAMETER	DESCRIPTION
OQ	**Open Quotient**The ratio of the fold open time to the total cycle time for a given position along the fold axis. The value of 100% means that the folds for a given point on their axis do not close the value 0% - which they do not open.
NCInsuf	**Non Closure Insufficiency** Coefficient describing the degree of regurgitation for a given point along the fold axis. It is counted only when regurgitation exists for a given point on the fold axis - it measures its level. It is determined as the percentage of the glottis width at the moment of maximum occlusion to the width at the moment of maximum opening. A value of 0% means almost the full range of fold movement, a value of 100% means no fold movement at all.

Table 5 Global parameters of the folds closing analyzer:

PARAMETER	DESCRIPTION
Non-closing (%FL)	**Non-closing part of vocal folds** Coefficient determining the fraction of folds (calculated as a percentage of the length along the axis) that do not close fully. It is the ratio of the number of graph points for which the OQ parameter is above 99% to the number of all graph points.
Non-opening (%FL)	**Non-opening parts of vocal folds**The coefficient of the fraction of folds (calculated as a percentage of the length along the axis) that do not open. It is the ratio of the number of plot points for which the OQ parameter is less than 1% to the number of all plot points.
EffectiveArea (%FL)	**Glottal effective area** Parameter defining the ratio of the active area of the glottis cavity to its maximum area. The active area of the glottis gap is defined as the difference between the minimum (when closed) and maximum (when opened) surfaces of the glottis.<br>EffecitiveArea = 100 – RGGA
RGGA (%FL)	**Relative Glottal Gap Area** Ratio of the minimum to maximum area of the glottis during the cycle.<br>RGGA = 100 – EffecitiveArea
OQAvg (%)	**Average Open Quotient** Average open time for folds. Averaged values for all points along their lengths and for their back 1/3 length, middle 1/3 length and front 1/3 length respectively.

The symmetry analyzer examines the movements of the vocal folds in a direction perpendicular to their axis. The level of asymmetry is determined. The analyzer is associated with one graph 25 with two data series (description in table 6) and global parameters presented in table 7. The unit of the X axis is percentages.

Table 6 Data series in an asymmetry analyzer graph:

PARAMETER	DESCRIPTION
AmplAsymmetry (%)	**Amplitude asymmetry** The factor compares the individual amplitude of the edge movements of both folds. It is calculated as the ratio of the absolute value of the difference between the individual amplitudes of the movement of both folds to the sum of the individual amplitudes: $AmplAsymmetry = abs(A_L - A_R) / (A_L + A_R)$ A value of 0% means that the resultant movement is an ideal combination of the movements of both folds (folds work fully symmetrically). A value of 100% means that there is no resultant movement of the folds (folds move in unison, one following the other). The value is determined for each point along the fold axis. The parameter is sensitive primarily to differences in the amplitude value, not very sensitive to differences in the phase of vibration of both folds.
PhaseAsymmetry (%)	**Phase asymmetry** The factor compares the sum of the individual amplitudes of the edge movements of both folds to the amplitude of their resultant movement. It is calculated as the ratio of the difference between the sum of individual amplitudes and the resultant amplitude to the sum of individual amplitudes: $AmplPhaseAsymmetry = (A_L + A_R - A_{LR}) / (A_L + A_R)$ A value of 0% means that the resultant movement is an ideal combination of the movements of both folds (folds work fully symmetrically). A value of 100% means that there is no resultant movement of the folds (folds move in unison, one following the other). The value is determined for each point along the fold axis. The parameter is sensitive primarily to differences in the vibration phase, not very sensitive to differences in amplitude.
Rel2CommonAmpl (%)	**Relative to common amplitude ratio** The coefficient determines the ratio of the amplitude of the virtual movement of the glottis center to the double of the resultant amplitude of their movement: $Rel2CommonAmpl = A_C/(2A_{LR})$ The calculated value is limited to 100%. The interpretation of the results is similar to the AmplAsymmetry parameter.

Table 7 Global parameters of the asymmetry analyzer:

PARAMETER	DESCRIPTION
AmplAsymAvg (%)	**Average amplitude asymmetry** The coefficient compares the individual amplitude of both vocal folds motion, according to the formula: AmpAsymmetry=(AmpL—AmpR)/(AmpL + AmpR)
PhaseAsymAvg (%)	**Average phase asymmetry** The average value of the AmplPhaseAsymmetry parameter for the folds. Averaged values for all points along their lengths and for their back 1/3 length, middle 1/3 length and front 1/3 length respectively.
AmplAsym-Weighted (%)	**Weighted amplitude asymmetry** Weighted average of the AmplAsymmetry parameter for whole folds (averaged values for all points along their length, including the weight variable for each point). The weight of each point included in the averaging is the amplitude of the resultant movement of the folds for that point along the folds.

PARAMETER	DESCRIPTION
Rel2CommonAmplAvg (%)	**Average relative to common amplitude ratio** Average value of the Rel2CommonAmpl parameter for whole folds (averaged values for all points along their length).

The phase difference analyzer examines the movements of the vocal folds in a direction perpendicular to their axis. The phase difference between the movements of the two folds is determined. The analyzer is associated with one graph 26 with one data series (description in table 6.8) and global parameters presented in table 6.9. The unit of the X axis is degrees. The phase difference is determined by describing both edges of the folds with periodic functions similar to sinusoids and determining the phase difference between these functions.

Table 8 Data series in a graph of a phase difference analyzer:

PARAMETER	DESCRIPTION
PhaseDiff (°)	**Phase difference**A parameter that determines the phase difference between the movement of both folds. An anatomical 180 ° difference is included and subtracted.

Table 9 Global parameters of the phase difference analyzer:

PARAMETER	DESCRIPTION
PhaseDiffAvg (°)	**Average phase difference** The average value of the phase difference (signed value, the sign indicates which of the vocal folds lags in phase) for the whole length of vocal folds and weighted value, respectively.
AbsPhaseDiffAvg (°)	**Average absolute phase difference** Average absolute phase difference value (unsigned value, the direction of the phase difference is ignored) for the whole length of vocal folds and weighted value, respectively.
Phase-DiffWeighted (°)	**Weighted phase difference** Weighted average value of the PhaseDiff parameter for whole folds (averaged values for all points along their length, including the weight variable for each point). The weight of each point included in the averaging is the amplitude of the resultant movement of the folds for that point along the folds).

The next tab is *Phonovibrogram* (Fig. 16). The phonovibrogram is a map presenting information about the movement of the vocal folds during the entire slow motion sequence. This graph provides information on the movement of the entire vocal folds over the entire time interval (not just one cross-section). When creating a phonovibrogram, information about the movement of the contours of the oscillating vocal folds is collected from the video sequence and then transformed into a monochrome phonovibrogram image.

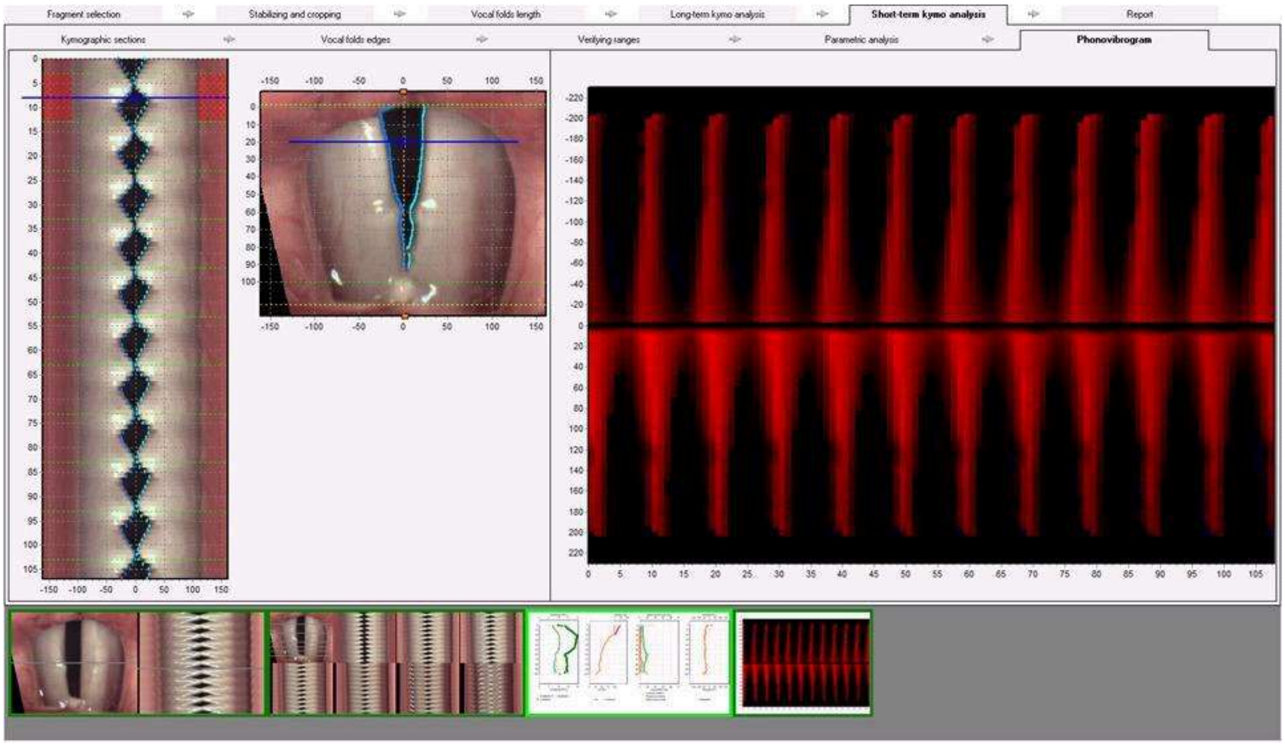

Fig. 16.Kymographic analysis window – Short-term kymo analysis -> Phonovibrogram

---

Next subject (*Table of Contents*) (../resources.html)

Copyright © 2022 DiagNova Technologies (http://www.diagnova.pl)
WS1_EU_20220708
